# Identification of compound causing yellow bone discoloration following *alpha*-glycosyl isoquercitrin exposure in Sprague–Dawley rats

**DOI:** 10.1007/s00204-020-02760-z

**Published:** 2020-05-09

**Authors:** Jeffrey P. Davis, Mihoko Koyanagi, Robert R. Maronpot, Leslie Recio, Shim-mo Hayashi

**Affiliations:** 1 Toxicology Program, Integrated Laboratory Systems, Inc., PO Box 13501, Research Triangle Park, NC 27709 USA; 2 Global Scientific and Regulatory Affairs, San-Ei Gen F.F.I., Inc., 1-1-11 Sanwa-cho, Toyonaka, Osaka 561-8588 Japan; 3 Maronpot Consulting LLC, 1612 Medfield Road, Raleigh, NC 27607 USA; 4 Division of Food Additives, National Institute of Health Sciences, 3-25-26 Tonomachi, Kawasaki-ku, Kawasaki, Kanagawa 210-9501 Japan

**Keywords:** Flavonol, *Alpha*-glycosyl isoquercitrin, AGIQ, EMIQ, Isoquercitrin, Quercetin, Bioanalysis, Toxicity, Sprague–dawley, Bone discoloration

## Abstract

Previous rat toxicity studies of *alpha*-glycosyl isoquercitrin (AGIQ), a water-soluble flavonol glycoside derived from rutin, revealed systemic yellow bone discoloration. This investigative study was conducted to determine the AGIQ metabolite(s) responsible for the discoloration. Female Sprague–Dawley rats were administered dietary AGIQ at doses of 0%, 1.5%, 3.0%, or 5.0% (0, 1735.0, 3480.8, and 5873.7 mg/kg/day, respectively) for 14 days, followed by a 14- or 28-day recovery period. Measurements of quercetin in urine and quercetin, quercetin 3-O-glucuronide, kaempferol, and 3-o-methylquercetin metabolites of AGIQ in bone (femur), white and brown fat, and cerebrum samples were conducted following the exposure period and each recovery period. Gross examination of the femur revealed yellow discoloration that increased in intensity with dose and was still present in a dose-related manner following both recovery periods. Quercetin, at levels correlating with AGIQ dose, was measured in the urine following the 14-day exposure period and, at lower concentrations, 14 or 28 days following cessation of AGIQ exposure. All four metabolites were present in a dose-dependent manner in the femur following 14 days of dietary exposure; only quercetin, quercetin 3-O-glucuronide, and 3-o-methylquercetin were present during the recovery periods. Quercetin, quercetin 3-O-glucuronide, and 3-o-methylquercetin were detected in white fat (along with kaempferol), brown fat (excluding quercetin due to analytical interference), and cerebrum samples, indicating systemic availability of the metabolites. Collectively, these data implicate quercetin, quercetin 3-O-glucuronide, or 3-o-methylquercetin (or a combination thereof) as the most likely metabolite of AGIQ causing the yellow discoloration of bone in rats administered dietary AGIQ.

## Introduction

Flavonoids such as the natural flavonol quercetin and its glycosides, including isoquercitrin (quercetin-3-*O-*D-glucoside) derived from rutin, are plant pigments found in many fruits and vegetables that have demonstrated potential benefits to human health, including reduction of inflammation, pain elimination, and cardiovascular protection (Amado et al. 2009; Gasparotto Junior et al. 2011; Kim et al. 2010; Li et al. 2011; Nyska et al. 2016; Valentova et al. 2014). These compounds, promoted as anti-oxidants, are available to consumers as dietary supplements. However, incorporation of natural quercetin glycosides into food and beverage products has been hampered by poor miscibility in water and limited absorption (Hobbs et al. 2018).

**Fig. 1:**
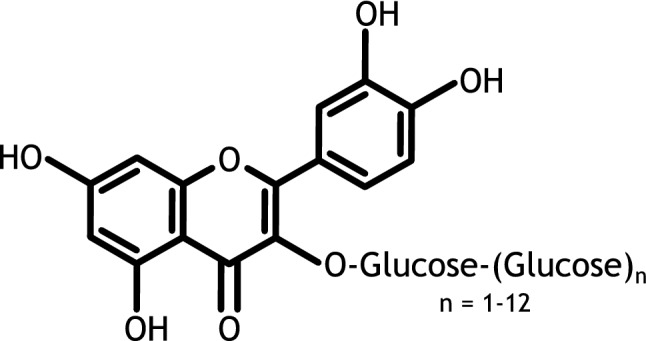
Chemical formula of *alpha*-glycosyl isoquercitrin

Enzymatic conjugation of multiple glucose moieties to isoquercitrin to produce *alpha*-glycosyl isoquercitrin (AGIQ, see Fig. 1), also called enzymatically modified isoquercitrin (EMIQ), has been shown to enhance solubility and bioavailability (Erlund et al. 2000; Manach et al. 1997). Commercial quantities of AGIQ are produced by glucosylating a mixture of isoquercitrin (enzymatically decomposed rutin from *Sophora japonica*, the Japanese pagoda tree) and dextrin with cyclodextrin glucanotransferase.

AGIQ is used in Japan as an additive in various beverages and foods. AGIQ was developed in 1987 and approved by the Japanese Ministry of Health and Welfare (MHW) for use as a food additive in 1996 (MHW 2014). Based on AGIQ’s favorable safety profile, the Expert Panel of the Flavor and Extract Manufacturers Association (FEMA) concluded that it was a generally recognized as safe (GRAS) compound in 2005 (Smith et al. 2005). The U.S. Food and Drug Administration (US FDA) also granted a GRAS status for AGIQ (GRN 220) as an anti-oxidant (US FDA 2007).

Previous toxicology and clinical studies of AGIQ have generally indicated that the compound is safe, non-carcinogenic, and non-genotoxic (Hasumura et al. 2004; Hobbs et al. 2018; Ishikura et al. 2008; Nyska et al. 2016; Salim et al. 2004; Tamano et al. 2001; Yoshimura et al. 2008). However, some of the older studies were not GLP compliant and/or used AGIQ of low or not fully detailed purity (Hasumura et al. 2004; Salim et al. 2004; Tamano et al. 2001; Valentova et al. 2014). Based on increased marketing of AGIQ to the consumer food and beverage industry, a recent series of GLP-compliant studies of high-purity AGIQ was undertaken to supplement the existing toxicity database. In a genotoxicity assessment, AGIQ tested positive in a bacterial reverse mutation assay, but was negative in the in vitro mammalian micronucleus and chromosomal aberration assays, micronucleus, and comet assays in male and female B6C3F1 mice and Sprague–Dawley rats, and Muta™ Mouse mutation assays that assessed multiple potential target tissues (Hobbs et al. 2018). A 90-day subchronic dietary toxicity study in Sprague–Dawley rats resulted in a no-observable-adverse-effect level (NOAEL) of 5% AGIQ in the diet (3461 and 3867 mg/kg/day for males and females, respectively) (Nyska et al. 2016).

Despite the general lack of toxicity, several studies of AGIQ or its metabolites have revealed the presence of yellow pigmentation, often in bones or the gastrointestinal (GI) tract mucosa (NTP 1992; Nyska et al. 2016; Tamano et al. 2001). Systemic dose-dependent yellow discoloration of all examined bones (femur, calvarium, and maxilla) was noted in AGIQ-exposed animals at all dose levels in the recent 90-day toxicity study, without discoloration of the GI tract mucosa (Nyska et al. 2016). There were no correlative microscopic changes in this study, and the yellow bone discoloration was considered toxicologically insignificant (Nyska et al. 2016). An older 13-week dietary toxicity study of enzymatically modified isoquercitrin in F344/DuCrj rats also resulted in yellow bone discoloration at doses up to 2.5% (highest dose tested) without corresponding histopathological changes or GI tract discoloration; the pigmentation was considered toxicologically negligible (Tamano et al. 2001). In a dietary 2-year carcinogenicity study evaluating quercetin in F344 rats, there was no observed evidence of carcinogenesis, but accumulation of yellow–brown granular pigment absorbed to or by the epithelial cells in the gastrointestinal (GI) tract was observed without any reported bone discoloration (NTP 1992).

The current dose–response and recovery study using highly purified AGIQ and Sprague–Dawley rats was conducted to identify the AGIQ metabolite causing yellow bone discoloration. Although this investigational study was not intended for submittal to any regulatory agency and, therefore, was not formally audited by the Integrated Laboratory Systems (ILS), Inc. Quality Assurance Unit, it was conducted to the highest standards of research practice, including quality control review of all collected data.

## Materials and methods

### Test article

The test article, AGIQ (> 97% pure, 0.13% quercetin, lot no. 170727), was provided by San-Ei Gen F.F.I., Inc., Osaka, Japan, as a yellow to yellowish-orange powder. AGIQ was blended into rodent chow for administration to the test animals.

### Animal husbandry and maintenance

Female Hsd:Sprague–Dawley^®^SD^®^ rats (obtained from Envigo, Frederick, MD) were assigned to the study. In the recent 90-day rat toxicity study, the severity of bone discoloration was greater in female Sprague–Dawley rats than in males (Nyska et al. 2016); therefore, female rats of the same strain were used as the test system in the current study.

The animals were 4–6 weeks of age at initiation of dose administration. The rats were allowed a 7-day period of acclimation to the facility conditions prior to inclusion in the study. The test article carrier diet, Purina Certified 5002 Meal Diet (Ralston Purina Co., St. Louis, MO) was offered ad libitum during acclimation and the AGIQ exposure period. Purina Certified 5002 Pelleted Diet (Ralston Purina Co., St. Louis, MO) was offered ad libitum following AGIQ exposure. The animals were allowed free access to drinking water (reverse osmosis-treated municipal tap water from the City of Durham, NC, analyzed annually by National Testing Laboratories, Inc., Cleveland, OH), supplied to each cage via polycarbonate water bottles equipped with stainless steel sipper tubes, throughout the study. Water bottles were changed at least once per week. All animals were housed singly during acclimation and AGIQ exposure and 2–3 per cage during the recovery period in polycarbonate cages with micro-isolator tops. Cages were changed at least twice weekly. Absorbent heat-treated hardwood bedding (Northeastern Products Corp., Warrensburg, NY) was provided and changed once per week. All animals were maintained on a 12-h daily photoperiod at an environmental temperature of 20–25 °C and relative humidity of 30–70%. Contaminant-screened polycarbonate enrichment tubes (Certified Rat Tunnels™, Bio Serv, Flemington, NJ) were provided to each animal.

The study was approved by the ILS, Inc. (Research Triangle Park, NC, USA) Animal Care and Use Committee, all procedures were in compliance with the Animal Welfare Act Regulations (9 CFR 1–4), and animals were handled and treated according to the Guide for the Care and Use of Laboratory Animals (ILAR 2011). The animal facilities at ILS, Inc. are Good Laboratory Practices (GLP)-compliant and accredited by the Association for Assessment and Accreditation of Laboratory Animal Care International (AAALAC International, Frederick, MD).

### Experimental design

Prior to the main dose–response/recovery study, a 14-day pilot study was conducted under the same conditions to develop analytical methods and identify potential metabolites in rat urine and femur. Three female rats per group were exposed to 5% AGIQ or the control diet for 14 days. Urine was collected daily and bilateral femurs were collected at termination. Metabolite profiling of the urine and bone (femur) identified several metabolites; quercetin and/or kaempferol were hypothesized to be the most likely candidates causing the yellow discoloration. Analysis of daily urine samples from rats exposed to 5% AGIQ demonstrated measurable concentrations of quercetin, whereas quercetin was not detected in control rats. Similarly, quercetin was detected in bone samples collected from animals exposed to 5% AGIQ, but not in bones of the control animals.

Based on these results, 60 female rats were randomized for the dose–response/recovery study into four exposure groups (15 animals/group) using a procedure that stratified animals across groups by body weight. The mean body weight of each group was not statistically significantly different from any other group using an analysis of variance (ANOVA, Statistical Analysis System, version 9.2, SAS Institute, Cary, NC). All animals allocated to the study were clinically healthy and weighed 91.1–111.6 g at initiation of dose administration.

Animals in Groups 1, 2, 3, and 4 were administered AGIQ in the carrier diet at dose levels of 0%, 1.5%, 3.0%, or 5.0%, respectively, for 14 consecutive days (Table 1). The doses and route of administration selected in this study were based on the gross observations of yellow bone discoloration in Sprague–Dawley rats administered AGIQ via the diet in the previous studies at dose levels greater than or equal to 1.5% following a 14-day exposure period (unpublished dose range-finding study) or 0.5% following a 90-day exposure period (Nyska et al. 2016). Following the 14-day exposure period, animals selected for recovery were provided the carrier diet alone. Five females/group/time point were euthanized after the 14-day exposure period or following a 14-day or 28-day recovery period.

**Table 1:** Study design

Dose group	Sex	AGIQ dose level (%)	No. of animals	Necropsy (days following 14-day exposure period)
1	F	0.0	5	0
			5	14
			5	28
2	F	1.5	5	0
			5	14
			5	28
3	F	3.0	5	0
			5	14
			5	28
4	F	5.0	5	0
			5	14
			5	28

*AGIQ* alpha-glycosyl isoquercitrin

### Viability, clinical signs, body weight, and food consumption

All animals were observed twice daily on weekdays and once daily on weekends and holidays for morbidity and mortality. Cage-side observations were conducted daily during the exposure period, and detailed clinical observations and body weight collections were performed on study days 1, 8, and 15, every 2 weeks thereafter, and prior to termination. Food consumption was measured weekly during the exposure period.

### Urinalysis

For urine collection, animals were maintained in metabolism cages overnight prior to euthanasia. An 18-h urine sample (kept cold during collection) was collected for each animal, dispensed into multiple aliquots with acetonitrile (MeCN, lot no. SHBJ6011, Sigma-Aldrich) at a 1:1 ratio to prevent solubility and absorption issues, and frozen at or below − 70 °C. One aliquot of urine per animal was analyzed for quercetin concentration using a previously qualified liquid chromatography with tandem mass spectrometry (LS/MS/MS) method in electrospray ionization (ESI) negative mode (API 5000 system, AB Sciex, Concord, Ontario) at Covance Laboratories, Inc. (Salt Lake City, UT).

### Necropsy and tissue handling

Abbreviated necropsies were performed on all animals following humane euthanasia (carbon dioxide asphyxiation confirmed by exsanguination) at the end of the exposure period and 14- and 28-day recovery periods. At necropsy, selected tissues (femur, maxilla, sternum, calvarium, white fat from the abdomen, brown fat from between the shoulder blades, and cerebrum) were examined for grossly visible lesions. Representative gross photographs of femurs were taken. Bilateral femurs (dissected in equal halves) and samples of white fat, brown fat, and cerebrum were collected, blown over with nitrogen, and frozen for bioanalysis.

### Tissue bioanalysis

Tissue samples collected at each necropsy (one femur sample per animal and samples of white fat, brown fat, and cerebrum) were analyzed for the presence of four metabolites of AGIQ (quercetin, quercetin 3-O-glucuronide, kaempferol, and isorhamnetin [3-o-methylquercetin]) using a validated UPLC-MS/MS method at the David H. Murdock Research Institute (DHMRI, Kannapolis, NC). The femur and white fat samples were analyzed on a Sciex Exion LC-X500R QTOF system (SCIEX, Framingham, MA), whereas the brown fat and cerebrum samples were analyzed on a Waters Acquity UPLC-Triple Quadrupole system (Waters Corp., Milford, MA) due to instability issues with these samples on the Sciex platform. Both systems were operated in ESI negative mode using mobile phases of 0.1% formic acid in water and 0.1% formic acid in acetonitrile. The raw data generated by the Waters UPLC-MS system were processed using the TargetLynx application manager (Waters Corp., Milford, MA), whereas the raw data generated by the Sciex UPLC-MS system were processed using Sciex Analytics.

### Statistical analysis

Group means and standard deviations were calculated and reported, with the following exception. Group median values were calculated and reported to summarize gross bone discoloration severity grades (Table 3). This study was investigational in nature and not intended for regulatory submission; therefore, no inferential statistical analyses were conducted on the data collected.

## Results

### Survival, clinical observations, body weight, and food intake

**Fig. 2:**
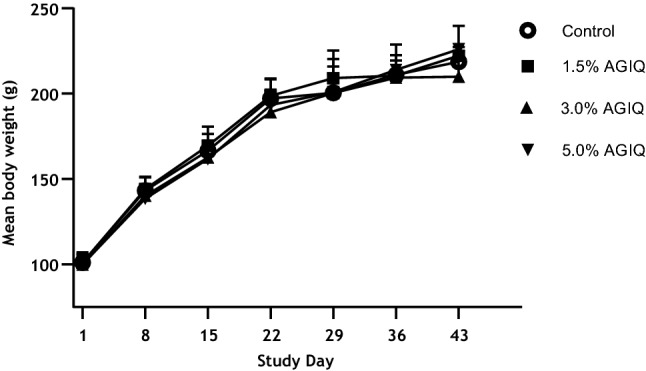
Growth curves of Sprague–Dawley rats administered dietary AGIQ at doses of 0%, 1.5%, 3.0%, or 5.0% for 14 days followed by 14 or 28 days of recovery

All animals survived to the scheduled euthanasia with no animals showing signs of moribundity. There were no AGIQ-related abnormal clinical or cage-side observations noted during the study. There were no significant changes in feed consumption, mean final body weight, or body weight gain in AGIQ-exposed rats when compared to concurrent controls. Mean body weights and feed/AGIQ consumption are presented in Fig. 2 and Table 2, respectively.

**Table 2:** Feed and test article consumption by Sprague–Dawley rats during 14-day AGIQ dietary exposure period

AGIQ dose level (%)	Sex	Mean feed consumption (g/kg body weight/day) ± SD (N)	Mean AGIQ consumption (mg/kg body weight/day) ± SD (N)
0.0	F	115.8 ± 5.18 (15)	NA
1.5	F	115.7 ± 3.61 (15)	1735.0 ± 53.9 (15)
3.0	F	116.0 ± 6.26 (15)	3480.8 ± 188.2 (15)
5.0	F	117.5 ± 5.68 (15)	5873.7 ± 284.4 (15)

*AGIQ* alpha-glycosyl isoquercitrin, *SD* standard deviation, *N* no. animals, *NA* not applicable

### Urine bioanalysis

**Fig. 3:**
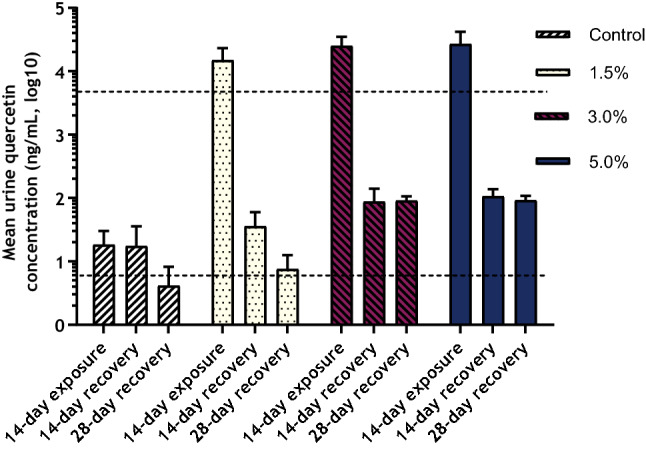
Mean quercetin concentrations in Sprague–Dawley rat urine following 14-day dietary AGIQ exposure or 14- or 28-day recovery period. Dotted lines indicate LLOQ (5.00 ng/mL) or ULOQ (5000 ng/mL)

Mean concentrations of quercetin in urine following the 14-day exposure and 14- and 28-day recovery periods are presented in Fig. 3.

There were measurable urine quercetin concentrations in the control animals at all collection time points. These values, which were near the lower limit of quantitation (LLOQ, 5.00 ng/mL), were similar across the three time points.

Following exposure to AGIQ for 14 days, urine quercetin concentrations in all test article-treated groups were above the upper limit of quantitation (ULOQ, 5,000 ng/mL). Values reported above the ULOQ were estimated via extrapolation of the standard curve. Quercetin concentrations in all AGIQ-exposed groups were decreased, but still present in concentrations above the LLOQ, following the conclusion of the 14- or 28-day recovery periods.

### Macroscopic observations

**Fig. 4:**
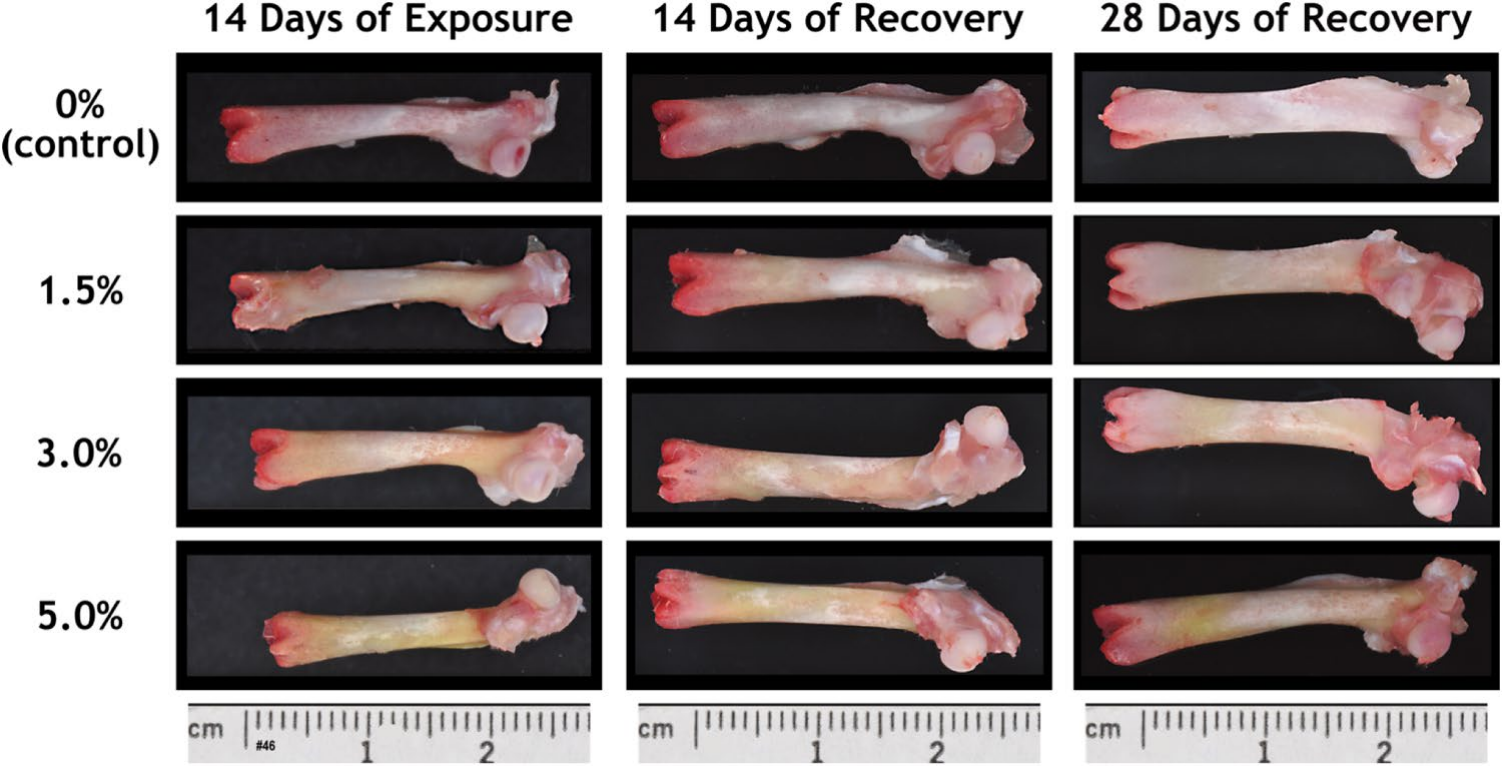
Macroscopic view of femurs from female Sprague–Dawley rats administered AGIQ in the diet at dose levels of 0%, 1.5%, 3.0%, or 5.0% for 14 consecutive days followed by 14 or 28 days of recovery

At necropsy, diffuse yellow discoloration of the femur was noted grossly in the AGIQ-exposed groups (Table 3 and Fig. 4). The median severity of the discoloration increased with increasing dose level at the end of the exposure period, with the exception of the high-dose group (due to one animal that was not recorded to have discolored femurs). It was considered likely that the severity of yellow discoloration for this single high-dose group animal was inadvertently not recorded. At the end of each recovery period, yellow discoloration of the femur was noted in the AGIQ-exposed groups at decreased median severity relative to the end-of-exposure time point, with the exception of the high-dose group value which remained the same. Similar yellow discoloration was also noted for the calvarium (data not presented).

**Table 3:** Gross femur discoloration severity in Sprague–Dawley rats administered dietary AGIQ

Necropsy time point	14-Day exposure				14-Day recovery				28-Day recovery			
AGIQ dose level	0%	1.5%	3.0%	5.0%	0%	1.5%	3.0%	5.0%	0%	1.5%	3.0%	5.0%
No. examined	5	5	5	5	5	5	5	5	5	5	5	5
No. with discoloration	0	5	5	4	0	5	5	5	0	5	5	5
Minimal	0	0	0	1	0	3	0	0	0	5	2	0
Mild	0	5	2	0	0	2	5	0	0	0	3	1
Moderate	0	0	3	3	0	0	0	5	0	0	0	4
Median Grade	0	2	3	3	0	1	2	3	0	1	2	3

*AGIQ* alpha-glycosyl isoquercitrin

Severity grades: None = 0; Minimal = 1; Mild = 2; Moderate = 3

### Tissue bioanalysis

Mean metabolite (quercetin, quercetin 3-O-glucuronide, kaempferol, and 3-o-methylquercetin) concentrations in femur, white fat, brown fat, and cerebrum tissues are presented in Table 4.

**Table 4:** Metabolite concentrations (mean ng/g tissue ± SD) in selected Sprague–Dawley rat tissues following 14 days of dietary AGIQ exposure or 14 or 28 days of recovery

Tissue	Metabolite	LLOQ	Time point	0% AGIQ	1.5% AGIQ	3.0% AGIQ	5.0% AGIQ
Femur	Quercetin	0.1864	14-day exposure	0.00 ± 0.00	3.61 ± 2.21	16.5 ± 2.26	35.7 ± 0.79
			14-day recovery	0.00 ± 0.00	0.22 ± 0.10	0.47 ± 0.18	0.95 ± 0.14
			28-day recovery	0.00 ± 0.00	0.06 ± 0.00	0.17 ± 0.03	0.11 ± 0.06
	Quercetin 3-O-glucuronide	0.4660	14-day exposure	0.00 ± 0.00	12.94 ± 7.40	43.39 ± 7.35	95.6 ± 2.49
			14-day recovery	0.00 ± 0.00	0.75 ± 0.32	4.05 ± 0.43	7.11 ± 0.22
			28-day recovery	0.00 ± 0.00	0.12 ± 0.08	0.55 ± 0.11	4.19 ± 0.11
	Kaempferol	1.8638	14-day exposure	0.00 ± 0.00	0.44 ± 0.00	4.16 ± 0.98	9.59 ± 0.98
			14-day recovery	0.00 ± 0.00	0.00 ± 0.00	0.00 ± 0.00	0.00 ± 0.00
			28-day recovery	0.00 ± 0.00	0.00 ± 0.00	0.00 ± 0.00	0.00 ± 0.00
	3-o-methylquercetin	0.1864	14-day exposure	0.01 ± 0.02	8.84 ± 5.29	54.62 ± 6.18	217.18 ± 3.37
			14-day recovery	0.00 ± 0.00	0.29 ± 0.11	0.63 ± 0.15	1.81 ± 0.07
			28-day recovery	0.00 ± 0.00	0.14 ± 0.05	0.28 ± 0.08	0.56 ± 0.02
White fat	Quercetin	4.4681	14-day exposure	0.00 ± 0.00	11.56 ± 9.41	120.45 ± 8.60	135.71 ± 7.00
			14-day recovery	0.00 ± 0.00	0.00 ± 0.00	1.13 ± 0.00	0.00 ± 0.00
			28-day recovery	0.00 ± 0.00	0.00 ± 0.00	0.00 ± 0.00	0.00 ± 0.00
	Quercetin 3-O-glucuronide	1.7873	14-day exposure	0.00 ± 0.00	3.46 ± 3.26	56 ± 3.22	166.07 ± 2.66
			14-day recovery	0.00 ± 0.00	0.00 ± 0.00	0.00 ± 0.00	0.00 ± 0.00
			28-day recovery	0.00 ± 0.00	0.00 ± 0.00	0.00 ± 0.00	0.00 ± 0.00
	Kaempferol	17.8725	14-day exposure	0.00 ± 0.00	0.00 ± 0.00	0.00 ± 0.00	2.72 ± 0.00
			14-day recovery	0.00 ± 0.00	0.00 ± 0.00	0.00 ± 0.00	0.00 ± 0.00
			28-day recovery	0.00 ± 0.00	0.00 ± 0.00	0.00 ± 0.00	0.00 ± 0.00
	3-o-Methylquercetin	0.4468	14-day exposure	0.63 ± 1.16	14.36 ± 11.96	76.37 ± 10.87	120.5 ± 7.44
			14-day recovery	0.03 ± 0.04	0.29 ± 0.02	0.57 ± 0.63	0.22 ± 0.63
			28-day recovery	0.00 ± 0.00	0.002 ± 0.00	0.00 ± 0.00	0.00 ± 0.00
Brown fat	Quercetin	2.0719	14-day exposure	0.00 ± 0.00	0.00 ± 0.00	0.00 ± 0.00	0.00 ± 0.00
			14-day recovery	0.00 ± 0.00	0.00 ± 0.00	0.00 ± 0.00	0.00 ± 0.00
			28-day recovery	0.00 ± 0.00	0.00 ± 0.00	0.00 ± 0.00	0.00 ± 0.00
	Quercetin 3-O-glucuronide	5.1796	14-day exposure	0.00 ± 0.00	2.27 ± 1.94	105.89 ± 2.41	214.07 ± 2.41
			14-day recovery	0.00 ± 0.00	0.00 ± 0.00	0.00 ± 0.00	0.00 ± 0.00
			28-day recovery	0.00 ± 0.00	0.00 ± 0.00	0.00 ± 0.00	0.00 ± 0.00
	Kaempferol	20.7185	14-day exposure	0.00 ± 0.00	0.00 ± 0.00	0.00 ± 0.00	0.00 ± 0.00
			14-day recovery	0.00 ± 0.00	0.00 ± 0.00	0.00 ± 0.00	0.00 ± 0.00
			28-day recovery	0.00 ± 0.00	0.00 ± 0.00	0.00 ± 0.00	0.00 ± 0.00
	3-o-methylquercetin	0.5180	14-day exposure	0.00 ± 0.00	4.42 ± 0.00	57.92 ± 3.41	109.56 ± 3.11
			14-day recovery	0.00 ± 0.00	0.00 ± 0.00	0.00 ± 0.00	0.00 ± 0.00
			28-day recovery	0.00 ± 0.00	0.00 ± 0.00	0.00 ± 0.00	0.00 ± 0.00
Cerebrum	Quercetin	0.3571	14-day exposure	0.00 ± 0.00	0.00 ± 0.00	0.79 ± 0.00	3.32 ± 0.00
			14-day recovery	0.00 ± 0.00	0.00 ± 0.00	0.00 ± 0.00	0.00 ± 0.00
			28-day recovery	0.00 ± 0.00	0.00 ± 0.00	0.00 ± 0.00	0.00 ± 0.00
	Quercetin 3-O-glucuronide	1.4286	14-day exposure	0.00 ± 0.00	0.00 ± 0.00	10.92 ± 0.00	18.25 ± 0.00
			14-day recovery	0.00 ± 0.00	0.00 ± 0.00	0.00 ± 0.00	0.00 ± 0.00
			28-day recovery	0.00 ± 0.00	0.00 ± 0.00	0.00 ± 0.00	0.00 ± 0.00
	Kaempferol	1.4286	14-day exposure	0.00 ± 0.00	0.00 ± 0.00	0.00 ± 0.00	0.00 ± 0.00
			14-day recovery	0.00 ± 0.00	0.00 ± 0.00	0.00 ± 0.00	0.00 ± 0.00
			28-day recovery	0.00 ± 0.00	0.00 ± 0.00	0.00 ± 0.00	0.00 ± 0.00
	3-o-methylquercetin	0.1429	14-day exposure	0.00 ± 0.00	2.36 ± 1.40	7.47 ± 1.77	10.71 ± 1.23
			14-day recovery	0.00 ± 0.00	0.00 ± 0.00	0.00 ± 0.00	0.00 ± 0.00
			28-day recovery	0.00 ± 0.00	0.00 ± 0.00	0.00 ± 0.00	0.00 ± 0.00

*AGIQ* alpha-glycosyl isoquercitrin; *SD* standard deviation; LLOQ lower limit of quantitation in ng/g tissue based on the average tissue mass (values below the LLOQ were extrapolated from the standard curve)

In the femurs of animals exposed to AGIQ for 14 days, quercetin, quercetin 3-O-glucuronide, kaempferol, and 3-o-methylquercetin were measured in an increasing dose-dependent manner. Quercetin, quercetin 3-O-glucuronide, and 3-o-methylquercetin were also observed in measurable but decreasing concentrations following the 14- and 28-day recovery periods. Kaempferol was not detected in the femur during either recovery period.

In white fat samples, quercetin, quercetin 3-O-glucuronide, and 3-o-methylquercetin were present in increasing levels in all animals as dose increased following the 14-day exposure period, whereas only one animal in the high-dose group was found to have a detectable concentration of kaempferol at the end of the exposure period. After the 14-day recovery period, quercetin was measured in one animal in the mid-dose group and 3-o-methylquercetin was measured in one to four animals per dose group. Following the 28-day recovery period, 3-o-methylquercetin was measured in one animal in the low-dose group. Kaempferol was not detected in any white fat samples at either recovery time point.

In brown fat samples, quercetin 3-O-glucuronide and 3-o-methylquercetin concentrations increased concurrently with increased dose level following the 14-day exposure period; these metabolites were not detected at the 14- or 28-day recovery evaluations. Quercetin concentrations were not able to be obtained for brown fat samples due to unknown and unresolved interference with the samples on two instrument platforms. No measurable concentrations of kaempferol were obtained in brown fat samples at any dose level or time point.

In the cerebrum of animals exposed to AGIQ for 14 days, quercetin and quercetin 3-O-glucuronide (both at dose levels of 3.0% and 5.0%), and 3-o-methylquercetin at all dose levels, were measured at increasing dose-dependent concentrations. None of these metabolites were detected at the recovery evaluations. Kaempferol was not detected in cerebrum samples at any dose level or time point.

## Discussion

The primary purpose of this study was to determine the AGIQ metabolite responsible for the yellow bone discoloration previously noted in dietary exposure studies of AGIQ in rats (Nyska et al. 2016; Tamano et al. 2001), which has also been noted in minipig studies of AGIQ (Maronpot et al. 2019). The absence of effects on survival, clinical condition, body weight, and food consumption and the presence of dose-dependent yellow femur discoloration at all dose levels in this study generally corroborated the results reported for those studies, with the exception of the decreased body weights observed in female rats at a dose level of 2.5% in one study (Tamano et al. 2001). As noted previously, there were no correlative histopathologic changes associated with the grossly observed yellow bone discoloration noted in the previous studies, and those findings were considered toxicologically insignificant (Nyska et al. 2016) or negligible (Tamano et al. 2001). Therefore, histopathology evaluations were not conducted in the current investigational studies.

Urine bioanalysis revealed detectable concentrations of the quercetin metabolite in urine following the exposure period and, at lesser concentrations, following both recovery periods. Quercetin was also detected in control rat urine at each time point during the dose–response/recovery study. Six control urine samples during the pilot study also had measurable quercetin concentrations above the LLOQ (10 ng/mL in that study). The reason for the presence of quercetin in the urine of control rats is unknown, but was likely due to potential low levels of quercetin already present in several vegetable components of the rodent chow (Purina Certified 5002 Diet) offered ad libitum in these studies. Quercetin is one of the most common flavonoids naturally present in a wide variety of fruits and vegetables (David et al. 2016; Hollman et al. 1997).

In contrast to the above-described results, quercetin was not detected in control blood samples during the toxicokinetic phase of the recent 90-day subchronic toxicity study of AGIQ, in which rats were housed at ILS under the same general husbandry conditions (Nyska et al. 2016). However, the rats in that study were maintained at ILS and given the Purina Certified 5002 Diet for only 3–4 days prior to collection of blood samples.

All four metabolites were present at dose-related concentrations in the femur following 14 days of dietary exposure, but only quercetin, quercetin 3-O-glucuronide, and 3-o-methylquercetin were present during the recovery periods (at decreased concentrations). Yellow discoloration of the femur following AGIQ exposure was most likely due to one or a combination of these three metabolites (quercetin, quercetin 3-O-glucuronide, and 3-O-methylquercetin), but a further study using direct dosing of animals with each metabolite individually is needed to provide additional clarity on the metabolite responsible for the yellow bone discoloration. The decreased severity of the discoloration and corresponding decreased concentrations of quercetin, quercetin 3-O-glucuronide, and 3-o-methylquercetin following 14 and 28 days of recovery indicated that the effect was likely completely reversible given a longer recovery period.

According to the GRAS notification provided to the U.S. FDA, AGIQ is intended for use as an anti-oxidant in multiple food categories at up to 150 mg/kg and in chewing gum at up to 1500 mg/kg (US FDA 2007). Specific examples of AGIQ-containing products in the Japanese market include bottled Japanese tea (listed as “Foods for Specified Health Uses”) containing 293 mg AGIQ (per 500 mL bottle) and a tablet (listed as “Foods with Function Claims”) containing 90 mg AGIQ/day (US FDA 2007). These intended and reported usage levels are well below the range of mean values for AGIQ consumption observed in the current study (1735.0 mg/kg/day in the 1.5% group to 5873.7 mg/kg/day in the 5.0% group).

The measurable presence of all four metabolites in white fat, two of the metabolites (quercetin 3-O-glucuronide and 3-o-methylquercetin) in brown fat, and three of the metabolites (quercetin, quercetin 3-O-glucuronide, and 3-o-methylquercetin) in cerebrum tissues indicated that these metabolites were systemically available. The systemic availability of AGIQ metabolites speaks well for the potential health benefits of AGIQ that were mentioned in the Introduction.
